# STAMBPL1 is a deubiquitinating enzyme that regulates HTLV-I Tax subcellular localization and NF-kB activation

**DOI:** 10.1186/1742-4690-8-S1-A190

**Published:** 2011-06-06

**Authors:** Alfonso Lavorgna, Edward W Harhaj

**Affiliations:** 1 Department of Microbiology and Immunology, Sylvester Comprehensive Cancer Center, University of Miami, Miller School of Medicine, Miami, FL, 33136, USA

## 

The human T-cell leukemia virus type 1 (HTLV-1) Tax oncoprotein is predominantly localized in the nuclei where it is associated with transcriptional and splicing regulatory proteins to form the Tax speckled structures (TSS). Tax is also distributed in the Golgi apparatus where it activates the IkB kinase (IKK) complex upstream of NF-κB, an essential transcription factor for Tax-induced immortalization of T lymphocytes. Lysine 63 (K63)-linked polyubiquitination of Tax is necessary for Tax to localize to the Golgi and activate NF-κB, whereas K48-linked polyubiquitination of Tax promotes its degradation in the nucleus. Therefore, post-translational modifications of Tax regulate its subcellular localization and hence its function, however the host factors that regulate Tax localization are unknown. Using a deubiquitinating (DUB) enzyme siRNA library we identified STAM-binding protein-like 1 (STAMBPL1) as a DUB that is required for Tax-mediated NF-κB activation. STAMBPL1 is a member of the JAMM (JAB1/MPN/MOV34 metalloenzyme) family of zinc metalloproteases, several of which have been previously implicated in the regulation of receptor endocytosis and trafficking. Overexpression of wild-type STAMBPL1, but not a catalytically inactive mutant, enhances Tax-mediated NF-κB activation and the induction of NF-κB target genes. Silencing of STAMBPL1 with siRNA inhibits Tax activation of both the canonical and noncanonical NF-κB signaling pathways. Furthermore, overexpression of STAMBPL1 enhances the stability of Tax by blocking its proteasomal degradation, whereas siRNA silencing of STAMBPL1 promotes Tax degradation. Confocal microscopy experiments reveal that STAMBPL1 controls the subcellular localization of Tax by promoting its nuclear export and subsequent trafficking to the Golgi where it activates IKK. Together these results suggest that the deubiquitinating enzyme STAMBPL1 regulates the localization and stability of Tax, and consequently the activation levels of NF-κB.

